# Caroli syndrome associated with atrial septal defect and polydactyly: a case report

**DOI:** 10.1186/s13256-023-03919-9

**Published:** 2023-05-23

**Authors:** Ali Ghassa, Lina Khouri

**Affiliations:** 1 Faculty of Medicine, Damascus University, Damascus, Syria; 2 Department of Gastroenterology, Children’s University Hospital, Damascus University, Damascus, Syria

**Keywords:** Caroli syndrome, ASD, Polydactyly, Splenomegaly

## Abstract

**Introduction:**

Caroli disease is multifocal segmental dilatation of the large intrahepatic bile ducts that connect to the main duct. It is considered a rare disease with an incidence rate of 1 in 1,000,000 births. There are two types of Caroli: the first type is the simple type, Caroli disease, which includes only cystic dilatation of the intrahepatic bile ducts. The second is called Caroli syndrome, which consists of Caroli disease and congenital hepatic fibrosis and might lead to portal hypertension leading to esophageal varices and splenomegaly. Atrial septal defect is one of the most common congenital heart diseases, occurring when the connection between the left and the right atriums fails to close. Polydactyly is one of the most common congenital malformations of the hands and feet. It manifests in excess fingers on the hands or toes.

**Case presentation:**

A 6-year-old Arab girl presented to the hospital with abdominal pain for the last month with abdominal enlargement. The patient was already diagnosed with Caroli disease and polydactyly (six fingers on each limb) when she was born. Investigations including complete blood count, blood smear, bone marrow biopsy, esophagoscopy, abdominal ultrasound, and computed tomography scan showed splenomegaly associated with hypersplenism, fourth-grade non-bleeding varices, intrahepatic cystic formations in the left and right lobes, and an atrial septal defect with a left-to-right shunt. The patient was scheduled for a splenectomy after she was vaccinated with the appropriate vaccines. After follow-up for a week in the hospital, complete blood count showed an improvement. A month after that, the patient had liver abscesses and biliary fistula that were treated appropriately and her symptoms resolved.

**Conclusion:**

The association of liver diseases, polydactyly, and congenital heart diseases is extremely rare and was only documented few times in the literature. However, to our knowledge, atrial septal defect has never been part of this combination before. The family history also makes this case unique and strongly suggests genetic etiology.

## Introduction

Caroli disease is multifocal segmental dilatation of the large intrahepatic bile ducts that connect to the main duct [1, 2]. It involves all the liver or just a lobe or segment [2]. It is considered a rare disease with an incidence rate of 1 in 1,000,000 births and was described for the first time by a French doctor named Caroli in 1958 [3, 4]. Caroli disease is categorized under the V group of Todani classification of fibrocystic biliary ducts diseases [4, 5]. There are two types of Caroli: the first type is the simple type, Caroli disease, which includes only cystic dilatation of the intrahepatic bile ducts. The second is called Caroli syndrome, which consists of Caroli disease and congenital hepatic fibrosis and might lead to portal hypertension [2, 3]. More than 80% of cases occur before the age of 30 [1].

We present an extremely rare case of a 6-year-old girl diagnosed with Caroli syndrome that is associated with atrial septal defect (ASD) and polydactyly and complicated with splenomegaly. To our knowledge, there is no such case in the literature combining all these abnormalities and that has a remarkable family history. However, there are few cases found to be similar to this.

## Case presentation

A 6-year-old Arab girl presented to the hospital with abdominal pain for the past month with abdominal enlargement. The patient was already diagnosed with Caroli disease when she was born. She was treated with medications in addition to a routine liver check-up every 3 months since she was born until now. Besides that, she has an atrial septal defect (ASD) discovered at the age of 8 months. She had an exploratory laparotomy with liver biopsy when she was 15 days old, which confirmed the diagnosis of Caroli disease, in addition to the removal of extra fingers at the age of 2 years due to polydactyly (six fingers on each limb). The patient is being treated with ursodeoxycholic acid 250 mg twice every day, ergocalciferol (sterogyl) injection every 3 months, vitamin K injection every 15 days, spironolactone 25 mg twice a day, propranolol 10 mg once a day since last year, and multivitamins once a day. The family history is remarkable; she has two deceased sisters. The first one died at the age of 1 month and had univentricular heart, unknown hepatic lesion, and polydactyly, and the second one died at the age of 1.5 years and had ASD and biliary atresia with Kasai procedure at the age of 2 months. There are no gastrointestinal or hematological diseases to be mentioned in the family. In addition, the parents are cousins. The mother declared that the child was thriving well except for starting to walk at the age of 3 years.

**Fig. 1:**
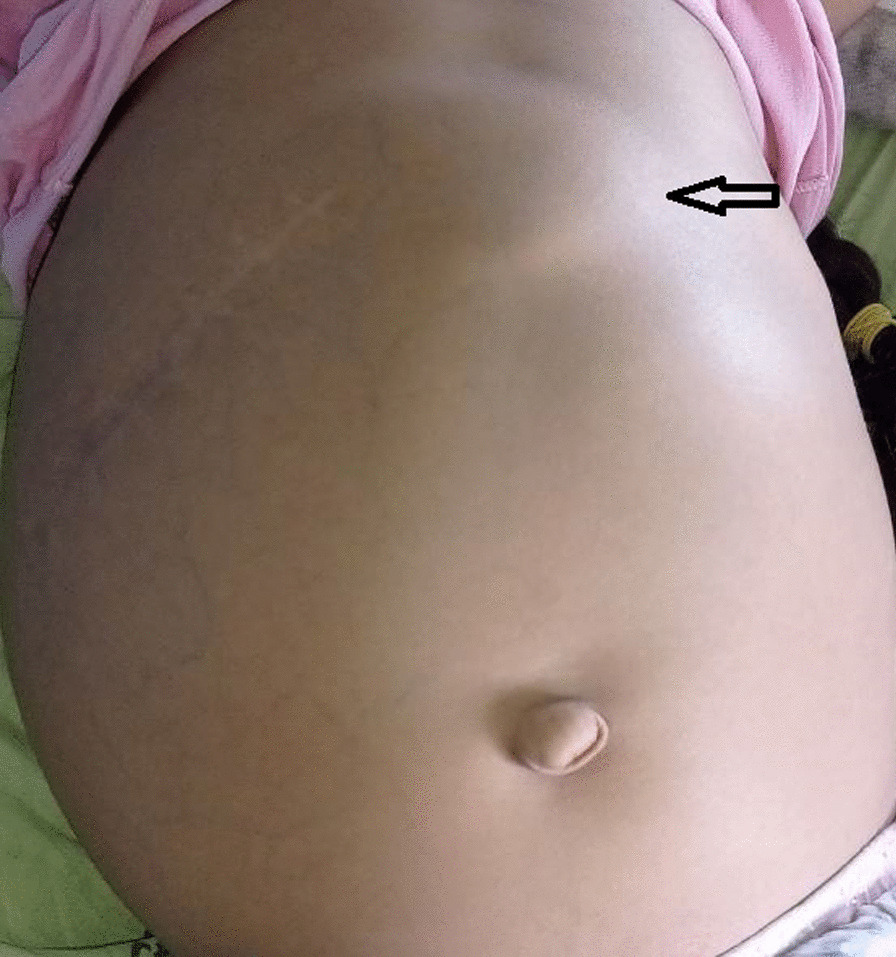
Picture of the distended abdomen showing splenomegaly (arrow)

The patient’s physical examination showed brown facies, icterus in the sclera and the skin, mild pallor, hirsutism, white nails, and clubbing of the fingers and toes. The abdomen showed an enlargement and no ascites was found (Fig. 1). The percussion of the abdomen was regular except for dullness over the spleen. The palpation showed splenomegaly measuring 10 cm in the mid-clavicle line. The auscultation revealed rough respiratory sounds and a systolic murmur rated 2 out of 6. Full neurological examination including motor function, sensation, reflexes, balance and coordination, cranial nerves, autonomic nervous system, and mental status was normal. Vital signs were measured on admission: (heart rate 95 beats per minute, respiratory rate 20 breaths per minute, temperature 37 °C).

**Fig. 2:**
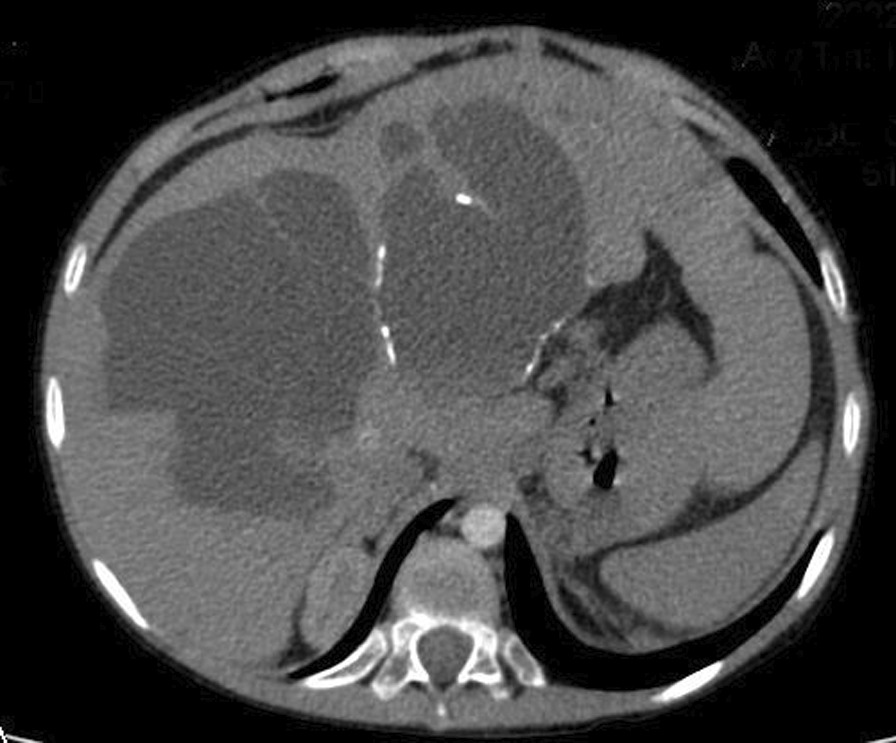
Abdominal CT scan with contrast showing intrahepatic cystic formations

**Fig. 3:**
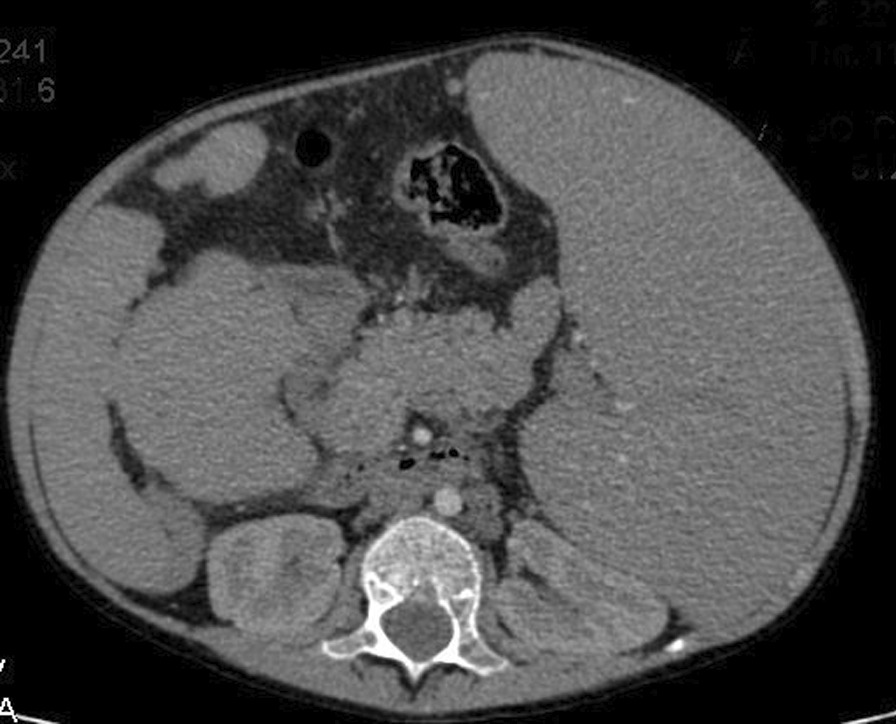
Abdominal CT scan with contrast showing splenomegaly

**Fig. 4:**
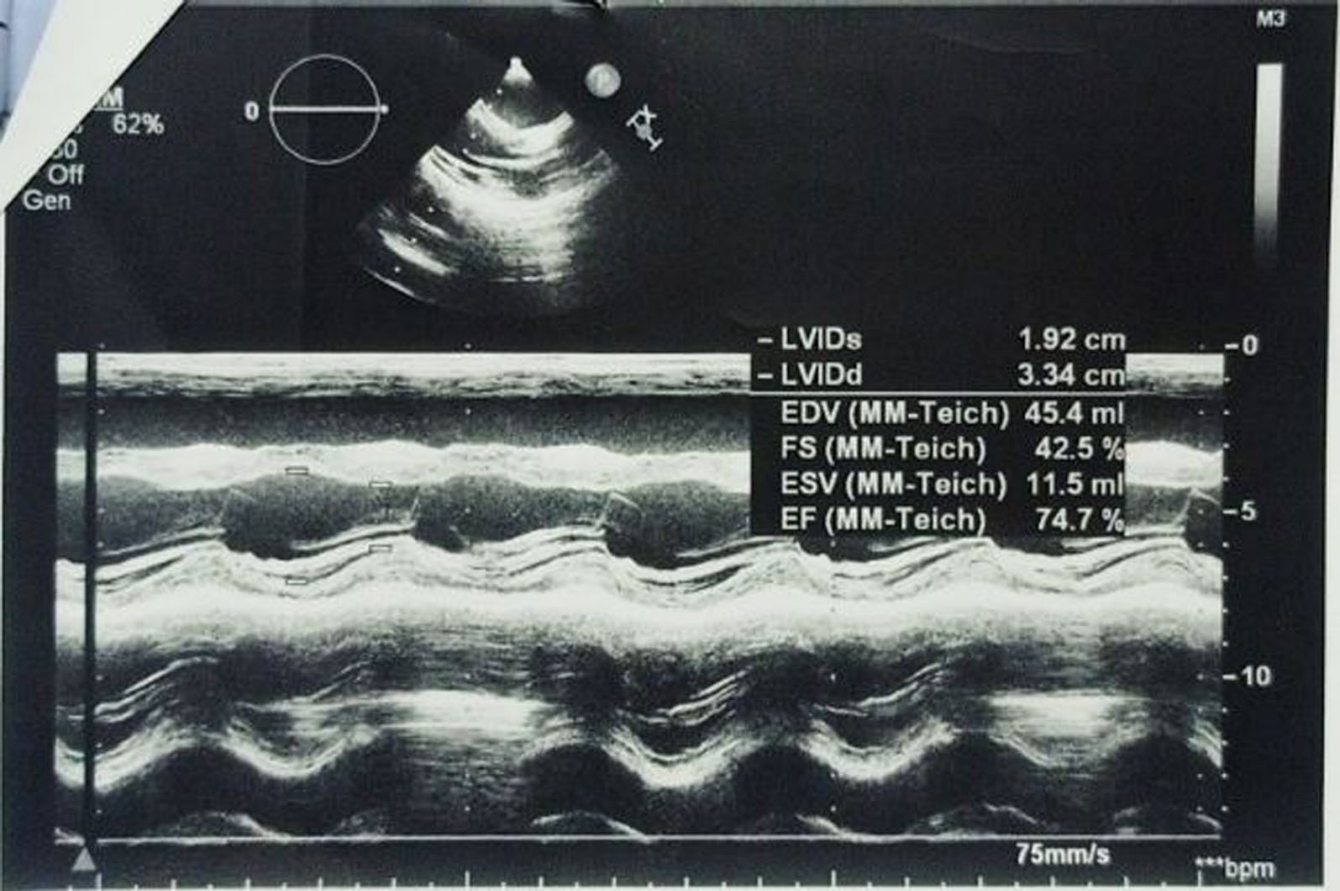
Cardiac ultrasound

The investigations started with laboratory tests (Table 1). Complete blood count (CBC) and blood smear showed pancytopenia. However, the bone marrow biopsy was normal. Esophagoscopy used to be done every 3 months for the past year. In the last one, fourth-grade non-bleeding varices were found. The abdominal ultrasound demonstrated multiple septated cystic formations within the liver containing turbid liquid. It also showed splenomegaly that measured 18 cm. The portal vein diameter was 7 mm, and the splenic vein measured 6 mm. Abdominal computed tomography (CT) scan with contrast showed intrahepatic cystic formations in the right and left lobes (Fig. 2). The greatest one measured 7 × 9.5 cm. These cysts have a tubular appearance and connect to the bile ducts inside the liver, which is consistent with Caroli disease. The liver borders are irregular with a cirrhotic look. There is a generalized enlargement of the spleen measuring 17 cm (Fig. 3). The kidney, the bladder, and the pancreas are normal. The cardiac ultrasound showed a secundum atrial septal defect (ASD) measuring 11 mm with left-to-right shunting and mild right heart dilatation. The pulmonary flow was 1.8 mm/second. Ejection fraction (EF) was 74%. (Fig. 4) In addition, no genetic study was carried out of the patient and her deceased siblings previously. The final diagnosis for the recent condition was splenomegaly with hypersplenism.

**Table 1:** Laboratory tests

Laboratory test	Test result	Normal range
Hemoglobin	9.8 mg/dl	11–14 mg/dl
MCV	90 fl	60–100 fl
Platelets	72.000/mm^3^	150.000–450.000/mm^3^
WBC	2100/mm^3^	4500–10,500/mm^3^
PT	70%	85–100%
PTT	44 second	25–35 second
Total bilirubin	23 mg/dl	0.1–1.2 mg/dl
Direct bilirubin	16 mg/dl	Less than 0.3 mg/dl
Albumin	3.1 g/dl	3.4–5.4 g/dl
AST	130 U/L	8–33 U/L
ALT	55U/L	4–36 U/L
Urea	16 mg/dl	6–24 mg/dl
Creatinine	0.2 mg/dl	0.3–0.6 mg/dl
Anti-HCV	0.21 (Negative)	Positive < 1.1
HBsAg	Negative	

*MCV* mean corpuscular volume,* WBC* white blood cells,* PT* prothrombin time,* PTT* partial thromboplastin time,* ALT* alanine transaminase,* AST* aspartate transaminase,* HBsAg* hepatitis B surface antigen

The patient was scheduled for splenectomy after receiving the pneumococcus, meningococcus, Haemophilus influenza and influenza vaccines. Splenectomy was done through a laparotomy without any complications. The pathology report of the spleen was congestion and splenic hyperplasia. She has been followed for 1 week in the hospital. Her blood test on day 7 came back as the following: hemoglobin 12.8 g/dl, MCV 90 fl, platelets 157.000/mm^3^, white blood count (WBC) 8500/mm^3^, total bilirubin 17.6 mg/dl, direct bilirubin 14.4 mg/dl, alanine transaminase (ALT) 112 U/L, aspartate aminotransferase (AST) 148 U/L. The patient presented the next month with fever and abdominal pain. Ultrasound and CT scan identified hepatic abscesses and biliary fistula. The patient was treated with antibiotics and drainage tube guided by ultrasound, after that, the symptoms resolved and the patient was discharged. The month after that, she also came in with fever, and a culture of the drainage showed *Klebsiella* and *E. coli*. She was treated with linezolid, tazocin, metronidazole, amikacin, and colistin. The drainage tube was also pulled out. Her symptoms resolved and lab tests returned normal.

## Discussion

This case presents a 6-year-old girl who came to the hospital with abdominal enlargement. She has a history of Caroli syndrome, ASD, and polydactyly. After investigations, she was diagnosed with splenomegaly, which was resolved by a splenectomy. This case is considered unique because, to our knowledge, there is no similar case in the literature that combines these three conditions. However, very few cases that combine liver fibrocystic diseases, congenital heart defects, and polydactyly were found to be similar to it. In addition, the family history of the patient makes the case even more exceptional.

Caroli disease is a rare condition characterized by saccular cystic segmental dilatation of the large intrahepatic bile ducts [1]. It has two forms; the first one is called Caroli disease (CD), which is pure cystic ectasia of the bile ducts that still connect to the biliary tree. However, the second form is called Caroli syndrome (CS), which is the ectasia of the bile ducts accompanied by congenital hepatic fibrosis or even liver cirrhosis, portal hypertension, and esophageal varices. [1, 2, 4].

The exact cause of this disease is not defined accurately, but the *PKHD1* gene, which is responsible for the synthesis of a protein called fibrocystin, is considered the cause. The protein formed after the expression of this gene, which is large and located on chromosome 6p12, plays a role in bile synthesis and the normal development of the liver and the kidney through controlling cell proliferation. Therefore, the total absence or abnormality of this protein causes massive damage to the liver parenchyma, seen as huge cystic formations [1]. A total of 520 mutations were discovered in the *PKHD1* gene, with 46 of them being the cause of CD [6]. In total, 30% of the patients who have these mutations die in the perinatal period because of respiratory failure caused by pulmonary hypoplasia, and the children who live develop kidney failure and portal hypertension [7].

Regarding the investigations, laboratory tests show elevated liver enzymes ALT and AST as well as elevated alkaline phosphatase (ALP) and direct bilirubin [1]. Radiological investigations are capable of diagnosing Caroli disease. Ultrasound is the best choice to start with because it is fast and cheap and demonstrates anechoic cystic formations in the intrahepatic bile ducts. It can also evaluate the kidney to search for any cystic kidney disease. However, ultrasound remains a weak diagnostic tool compared with other diagnostic tools. CT scan reveals an abnormally shaped liver with a “central dot” appearance, which is dark cystic dilatation around a central bright spot that contains branches of the portal vein and the portal hepatic artery. Magnetic resonance imaging (MRI) is as accurate as CT scan, but it can also diagnose Caroli disease in the prenatal period. Magnetic resonance cholangiopancreatography (MRCP) is considered the most sensitive and specific test, as it shows the connections between cystic formations and the normal biliary tract and helps to exclude other conditions such as multiple liver abscesses and polycystic liver disease. In addition, endoscopic retrograde cholangiopancreatography (ERCP) has a high sensitivity in diagnosing Caroli disease because it evaluates the entire biliary tree and helps in stone removal by sphincterotomy during cholangitis bouts. A liver biopsy is rarely done and can help diagnose liver fibrosis [1, 2, 5, 7].

In Caroli disease, there is biliary stasis that forms stones obstructing the bile ducts, so that patients suffer from recurrent cholangitis [1]. The most common symptom in CD is abdominal pain in the right upper quadrant. Other symptoms are high fever, anorexia, fatigue, and jaundice [2]. Manifestations of Caroli syndrome are caused because of liver cirrhosis and portal hypertension. The symptoms are splenomegaly, ascites, peripheral edema, coagulation disorders, and variceal bleeding. In addition, pancytopenia is seen in CS more commonly than in CD [2, 5]. Physical examination shows tenderness in the right upper quadrant of the abdomen, with a negative Murphy’s sign, hepatomegaly due to huge cysts, and scleral icterus [1].

The complications of Caroli are recurrent bouts of cholangitis and liver abscesses that can have antibiotic-resistant bacteria, which can cause lethal infection or secondary biliary cirrhosis. Chronic inflammation of the biliary tree increases the probability of cholangiocarcinoma by 100 times. In addition, portal hypertension can lead to esophageal varices and splenomegaly, which consequently can lead to pancytopenia. Death eventually occurs because of liver failure or portal hypertension complications such as variceal bleeding [1, 3, 4, 8, 9].

Treatment of Caroli disease is supportive and depends on the symptoms. For cholangitis, the treatment is antibiotics. ERCP is used for stone removal and stent insertion. Ursodeoxycholic acid is used to treat biliary stasis and reduce stone formation. For esophageal varices, beta blockers are used, and in case of bleeding, endoscopic ligation is the solution. Diuretics are also given to treat ascites [1, 8]. Liver resection is the primary treatment of unilobar disease in case of the absence of recurrent cholangitis, liver fibrosis, and cirrhosis. However, the diffuse hepatic disease that is complicated with liver fibrosis or portal hypertension should be treated with liver transplantation [10]. The prognosis of Caroli disease differs according to the accompanying genetic abnormality (deletion or mutation) and the number of affected organs [1].

Atrial septal defect (ASD) is one of the most common congenital heart diseases, occurring when the connection between the atriums fails to fuse. It has many types on the basis of its site. ASD is usually asymptomatic but the patient might present with exertional dyspnea and sometimes palpitation due to atrial fibrillation. The treatment depends on the size of the ASD [11, 12]. ASD and other congenital heart defects arise from a multifactorial origin with interaction of genetic and environmental factors [13]. Romero *et al*. mentioned a case of a man with autosomal recessive polycystic kidney disease who came to the hospital with abdominal pain, fever, and diarrhea in their paper. Physical examination showed splenomegaly, pancytopenia, and heart murmur. Transesophageal ultrasound revealed ASD, and a liver biopsy confirmed the diagnosis of Caroli. This is an example of the association between Caroli syndrome and ASD [14]. However, after research in the literature, we were not able to find any reasonable cause for this association.

Polydactyly is one of the most common congenital malformations of the hands and feet, manifesting as excess fingers and toes. The excess finger might only be a small part of a soft tissue or may be a completely physiologic finger. It might be in one limb or the four extremities, sporadic or part of a syndrome. It can be transmitted through autosomal recessive or dominant inheritance. Having four excess fingers and toes, which is called tetrapolydactyly, is considered very rare [15]. There are syndromes such as Ellis van Creveld that combine congenital heart defects and polydactyly in addition to other manifestations. This syndrome is associated with mutations in *EVC1* and *EVC2* genes on the short part of the fourth chromosome [16, 17]. Dogan *et al*. mentioned the association of primum ASD with tetrapolydactyly in their paper, but this association remains rare [16].

A study showed that decreased expression of the *TTC26* gene, which is located in the chromosome 7q34, can cause severe ciliopathies, and the phenotype includes cystic dilation of the intrahepatic biliary ducts and polydactyly in addition to other characteristics [18]. This might explain the association between Caroli and polydactyly.

In their paper, Esmer *et al*. described two cases mentioned for the first time in 2001 that involved the association between liver fibrocystic diseases, polydactyly, and congenital heart diseases. The first case was a combination of tetrapolydactyly, congenital liver fibrosis, and persistent ductal arteriosus (PDA), while the second case was a combination of tetrapolydactyly, Caroli syndrome, ventricular septal defect (VSD), and persistent ductal arteriosus (PDA) [19].

After research, we could not find any exact cause for these three entities together. There are many probabilities of this occurrence; it might have arisen by chance, or it might be a proposal of a new syndrome. In addition, the family history is significant due to the existence of both liver disease and congenital heart disease in the two deceased siblings and polydactyly in one of them, and since the parents are also cousins this strongly suggests genetic etiology in the three daughters. However, more research regarding this association is required.

## Conclusion

The association between liver diseases, polydactyly, and congenital heart diseases is extremely rare and was only documented a few times in the literature. However, to our knowledge, ASD has never been part of this combination before. The family history also makes this case unique and proposes genetic etiology.

## Data Availability

All data generated during this study can be accessed through direct communication with the corresponding author and the agreement of all research team members.
